# Progress and Market Development of Biotechnology in Saudi Arabia: A Survey -

**DOI:** 10.31661/gmj.vi.3964

**Published:** 2025-07-27

**Authors:** Asma Alyaemni, Nouf Alghamdi

**Affiliations:** ^1^ Health Administration, College of Business Administration, King Saud University, Saudi Arabia; ^2^ Molecular Business Unit, Zahrawi Group, Saudi Arabia

**Keywords:** Biotechnology, Saudi Arabia, Vision 2030, Regulatory Reforms, Healthcare Biotechnology, Agricultural Biotechnology, Industrial Biotechnology, Innovation

## Abstract

**Background:**

Biotechnology is a transformative field with applications in healthcare, agriculture, and industry. Saudi Arabia has prioritized biotechnology under Vision 2030, aiming to diversify its economy and establish itself as a regional biotech hub. Initiatives like the Saudi Genome Program and regulatory reforms by the SFDA highlight progress, yet challenges such as workforce shortages, import dependency, and ethical concerns persist. This study examines the perceptions of biotechnology professionals in Saudi Arabia regarding sectoral progress, regulatory efficiency, and market opportunities, focusing on genetic research, precision medicine, and industrial biotechnology under Vision 2030.

**Materials and Methods:**

A descriptive-analytical design was employed, targeting 150 biotechnology researchers, industry professionals, and policymakers via purposive sampling. Data were collected through a validated Likert-scale questionnaire assessing regulatory adequacy, collaboration, local manufacturing, and import challenges. Quantitative analysis was performed using SPSS v20.0.

**Results:**

While 40% agreed that SFDA regulations support genetic testing growth, 35.3% reported insufficient academia-industry-government collaboration. Optimism was high for local manufacturing (76.7%) and Saudi Arabia’s potential as a regional biotech hub (83.4%). However, 67.3% faced import barriers, and 87.3% noted delays hindering research. Genetic data privacy concerned 55.3%, while 90.7% endorsed global partnerships for innovation.

**Conclusion:**

Saudi Arabia’s biotechnology sector shows promise under Vision 2030, with strong potential in local manufacturing and regional leadership. However, regulatory harmonization, enhanced collaboration, and infrastructure investment are critical to overcoming import dependencies and workforce gaps. Strategic policy interventions are recommended to sustain growth and innovation.

## Introduction

Biotechnology is a multidisciplinary field that utilizes biological systems and living organisms to address challenges across sectors such as healthcare, agriculture, and industry. The global biotechnology market is expected to exceed $837 billion by 2030 [1]. These technologies have influenced a wide range of applications and have led to structural changes in various sectors. Countries with established biotechnology ecosystems have integrated academic research, regulatory policy, and commercial investment to foster product development and technological advancement [2]. These systems facilitate innovation through coordinated policies, streamlined regulation, and collaboration between public and private stakeholders. Saudi Arabia has identified biotechnology as a priority area in Vision 2030, which emphasizes economic diversification and the transition to a knowledge-based economy.

This focus is evident in recent policy reforms, infrastructure development, and funding allocations intended to support biotechnology research and commercialization [2][3]. National programs such as the Saudi Genome Program and planned biotechnology zones in NEOM illustrate the government’s ambition to establish the Kingdom as a regional biotechnology center [4].

Saudi Arabia’s biotechnology sector focuses on genomics, personalized medicine, and local pharmaceutical production, spanning healthcare (Saudi Genome Program, biopharmaceuticals), agriculture (CRISPR crops, vertical farming), and industry (biofuels, biodegradable materials) [5][6]. These efforts align with global trends in sustainable development [7] and are supported by regulatory frameworks from the SFDA, though challenges remain in harmonization and approval efficiency [8][9]. Vision 2030 drives growth in biopharmaceuticals and synthetic biology [10], while desert agriculture and halal bioproducts offer regional market opportunities [5]. However, constraints like skilled workforce shortages, import dependency, and limited private R&D investment require targeted policy interventions [7].

The literature highlights Saudi Arabia’s biotechnology expansion under Vision 2030 [2], emphasizing regulatory progress and the need for academic-industry collaboration [4]. Ethical concerns, such as genetic data privacy [11], and infrastructure gaps persist, alongside opportunities in localized manufacturing [10].

## Materials and Methods

This study employed a descriptive-analytical design to investigate perceptions, opportunities, and challenges associated with the biotechnology sector in Saudi Arabia. The research particularly emphasized developments in genetic research, precision medicine, and industrial biotechnology, all in the context of Saudi Arabia’s Vision 2030 initiative [2].

### Study Population and Sampling

The target population for this study consisted of biotechnology researchers, industry professionals, policymakers, and academic experts actively involved in the biotechnology and genetic research sectors within Saudi Arabia. A two-stage purposive sampling technique was utilized to ensure the inclusion of individuals with relevant expertise and experience. In the first stage, key institutions (King Abdullah International Medical Research Center, Saudi Biotechnology Society, and leading private biotech firms) were identified. In the second stage, participants were recruited based on their roles and contributions to the sector.

Inclusion criteria required participants to have at least one year of professional experience in biotechnology or genetics and a minimum qualification of a bachelor’s degree in a relevant field. Individuals not meeting these criteria, those working outside Saudi Arabia, or those in unrelated sectors (like agriculture without biotech applications) were excluded from the study.

Potential respondents were identified through professional networks (LinkedIn), contact numbers, institutional directories (Saudi Biotech Society), and government registries (Vision 2030 Biotechnology Task Force members). An electronic consent form was attached to the survey, outlining the study’s purpose, confidentiality measures, and voluntary participation. Only those who provided consent could proceed. Reminder was sent 7 days after initial distribution via WhatsApp, email, and LinkedIn.

Out of 220 invitations sent (accounting for potential attrition), 167 responses were received, while 17 partial submissions were excluded from the final analysis due to incomplete data, yielding final 150 fully completed responses (68% response rate).

### Data Collection and Instrumentation

Data were collected using a validated, structured, self-administered questionnaire developed specifically for this research, distributed to biotechnology professionals in Saudi Arabia. The questionnaire design was informed by an extensive review of the literature and strategic national documents, particularly Vision 2030 priorities for biotechnology [5]. The instrument, the survey, consisted of two main sections: the first collected demographic information, including gender, role, years of experience, field of expertise, and institution type; the second assessed perceptions on regulations, innovation, manufacturing, and import challenges in 7 questions as shown in Table-1. Participants rated their agreement with statements using a five-point Likert scale ranging from "Strongly Disagree" to "Strongly Agree." The reliability of the questionnaire was confirmed with a Cronbach’s alpha of 0.795, indicating good internal consistency.

### Data Analysis

The percentage of responses for each category was converted into weighted average scores per question by multiplying the response distribution by their respective numerical values and summing the results, with higher scores (closer to 5) indicating stronger endorsement and lower scores (closer to 1) reflecting disagreement, as shown below:

Average Score=(%Strongly disagree×1)+(%Disagree×2)+(%Neutral×3)+(%Agree×4)+(%Strongly agree×5)

Quantitative data were analyzed using SPSS v20.0 (IBM SPSS Statistics, IBM, USA), employing descriptive statistics (frequencies, means, standard deviations).

### Ethic Code

Ethical Approval for this study was obtained from the Sub-committee on Ethics

Human and Social Research. King Saud University (IRB No:596-25)

## Results

**Figure-1:**
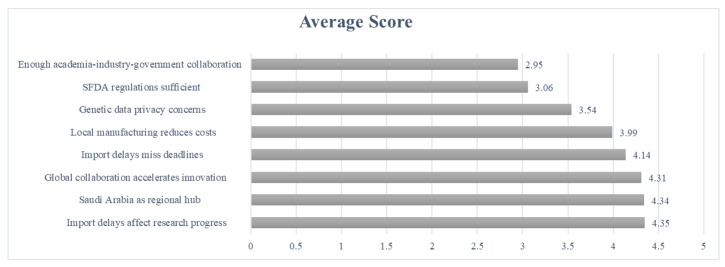
Figure 1. Likert Scale Responses (1–5) on Biotechnology Challenges and Opportunities in Saudi Arabia

**Table:** Table1. Questions of the Survey Used in Study

**number**	**Questionnaire Items **
1	Are current SFDA regulations sufficient to support the growth of genetic testing in Saudi Arabia
2	Is there enough collaboration between academia, industry, and government to advance genetic testing
3	Do you believe local manufacturing of genetic testing kits would significantly reduce costs for healthcare providers
4	Do you think Saudi Arabia can become a regional hub for genetic testing and precision medicine
5	Have you encountered challenges in importing genetic testing products or reagents due to regulatory constraints
6	Do you believe Saudi Arabia has the potential to develop and commercialize its own genetic testing kits for global markets
7	Is genetic data privacy and ethical management a major concern for the expansion of genetic testing in Saudi Arabia
8	Do you think increased collaboration between Saudi biotech companies and global genetic research institutions would accelerate innovation in the country
9	Have you experienced delays in receiving imported genetic testing materials in Saudi Arabia
10	Do you believe that importation delays significantly affect research progress in genetic testing
11	Have delays in importation led to missed research deadlines or grant timelines

A total of 150 participants responded to the survey, that the majority were male (64.7%, n=97), while 35.3% (n=53) were female. In terms of primary roles in biotechnology, most participants identified as researchers or end users (73.3%, n=110), followed by sales roles (9.3%, n=14), procurement (4.7%, n=7), technicians (3.3%, n=5), and application specialists (2%, n=3), with smaller proportions in quality assurance (2%, n=3), genetic counseling (1.3%, n=2), chief operations officers (1.3%, n=2), medical technologists (1.3%, n=2), and other roles (1.3%, n=2). Regarding years of experience, 80.7% (n=121) had over five years, 12% (n=18) had 1-3 years, and 7.3% (n=11) had 3-5 years. Expertise distribution included genomics (48%, n=72), clinical research (24.7%, n=37), and other fields (27.3%, n=41). Most participants worked in governmental institutions (80.7%, n=121), while 19.3% (n=29) were affiliated with private institutions.

### Survey Results

The survey reveals mixed perceptions regarding the sufficiency of current SFDA regulations to support genetic testing growth, with 35.3% of respondents neutral and 40% agreeing or strongly agreeing. Collaboration between academia, industry, and government appears limited, as 35.3% disagree that it is sufficient, while only 32% agree. However, optimism exists for local manufacturing, with 76.7% believing it could reduce costs, and 83.4% seeing Saudi Arabia as a potential regional hub for genetic testing. Despite this, regulatory challenges persist, with 67.3% reporting difficulties in importing genetic testing materials, and 87.3% stating that delays impact research progress.

Concerns about genetic data privacy remain notable, with 55.3% considering it a major issue. Meanwhile, strong support exists for global collaboration, as 90.7% believe partnerships with international institutions would accelerate innovation. Import delays are a significant hurdle, with 80.7% experiencing delays and 76% confirming these delays affect deadlines.

The highest agreement was observed for "Do you believe that importation delays significantly affect research progress in genetic testing?" (M=4.35, SD=0.91), followed closely by "Do you think Saudi Arabia can become a regional hub for genetic testing and precision medicine?" (M=4.34, SD=0.89) and "Do you think increased collaboration between Saudi biotech companies and global genetic research institutions would accelerate innovation in the country?" (M=4.31, SD=0.79). Conversely, the lowest agreement was reported for "Is there enough collaboration between academia, industry, and government to advance genetic testing?" (M=2.95, SD=1.12), indicating a perceived lack of interdisciplinary cooperation. Other notable findings included strong support for local manufacturing reducing costs (M=3.99, SD=0.98) and concerns over importation delays affecting deadlines (M=4.14, SD=0.94). detailed scores are shown in Figure-1.

**Figure-2:**
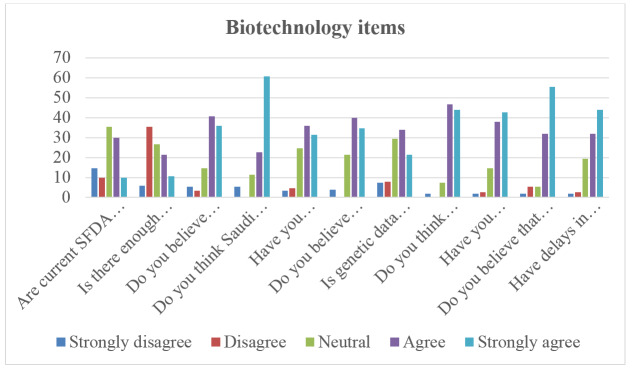
Figure 2. Biotechnology items

## Discussion

Saudi Arabia’s biotechnology sector is advancing under Vision 2030, with a focus on healthcare, genomics, and industrial biotechnology to establish the Kingdom as a regional biotech hub [2][7]. The sector benefits from a skilled workforce, with 73.3% of surveyed professionals actively engaged in research and 80.7% having over five years of experience. However, challenges persist, including regulatory inefficiencies, only 40% of respondents believe SFDA regulations adequately support genetic testing, and delays in biologics approvals, which take 14 months on average [7][8][12]. Collaboration between academia, industry, and government remains weak, with only 32% of respondents agreeing that partnerships are effective [4][13]. Import dependency also hampers progress, with 74% reporting delays in securing genetic testing materials, impacting research [14]. Despite these hurdles, 76.7% believe domestic production of testing kits could reduce costs, aligning with localization efforts like SaudiVax and SPIMACO initiatives [5].

Optimism about the sector’s potential is high, with 83.4% seeing Saudi Arabia as a future regional leader in precision medicine, supported by the Saudi Genome Program’s sequencing of 100,000 genomes [4]. However, ethical concerns over genetic data privacy (highlighted by 55.3% of respondents) and talent shortages, such as only seven certified bioinformatics professionals, pose significant barriers [11][9][12]. Heavy reliance on imports (94% of equipment, 89% of reagents) and regulatory delays further constrain growth, though foundational achievements like mRNA vaccine production offer promise. Addressing these challenges requires accelerated licensing, workforce development, and supply chain investments. With targeted reforms, Saudi Arabia aims for 5,000 annual patents and $5 billion in R&D by 2030, positioning itself as a key regional biotech player.

Recent studies highlight critical gaps and opportunities in biotechnology awareness, education, and industry growth across different contexts. Alanazi (2021) found limited knowledge of biotechnology among Saudi secondary students and teachers, with educators criticizing the insufficient coverage of biotechnology in science curricula [15]. Similarly, Uddin et al. [16] emphasized the importance of public perception in biotechnology acceptance, noting that successful commercialization depends on societal awareness and regulatory support. In Saudi Arabia, Prabhu [17] identified technological, financial, and governmental factors as pivotal for biotech firm success, aligning with Vision 2030 goals. However, challenges such as workforce shortages, import dependency, and weak academia-industry collaboration persist, despite optimism about local manufacturing and regional leadership potential (Author, Year). These findings collectively underscore the need for enhanced education, regulatory harmonization, and strategic investments to advance biotechnology innovation and public engagement.

## Conclusion

This study analyzes Saudi Arabia’s biotechnology sector, highlighting its growth driven by government initiatives and a skilled workforce, with optimism about leadership in genetic research and precision medicine. However, challenges such as regulatory delays, limited sector collaboration, and reliance on imports persist. The regulatory environment needs efficiency improvements and international alignment, while stronger academic-government-industry cooperation is essential for innovation. Stakeholders stress the need for domestic manufacturing, workforce development, and R&D investment to sustain growth. With a 72.15% perception score, the sector aligns with Vision 2030 goals, but achieving regional leadership requires policy reforms, investment, and international partnerships. Addressing these challenges can strengthen Saudi Arabia’s position as a biotech innovator.

## Conflict of Interest

The authors declare no competing interests.
